# Neonatologist-Performed Echocardiography in Neonatal Pulmonary Hypertension: A Narrative Review of the Literature

**DOI:** 10.3390/diagnostics15243154

**Published:** 2025-12-11

**Authors:** Anna Chiara Titolo, Mandy Ferrocino, Eleonora Biagi, Luisa Rizzo, Hajrie Seferi, Valentina Dell’Orto, Serafina Perrone, Susanna Esposito

**Affiliations:** 1 Pediatric Clinic, Parma University Hospital, 43126 Parma, Italy; annachiara.titolo@unipr.it (A.C.T.); mandy.ferrocino@unipr.it (M.F.); eleonora.biagi@unipr.it (E.B.); luisa.rizzo@unipr.it (L.R.); hajrie.seferi@unipr.it (H.S.); 2 Department of Medicine and Surgery, University of Parma, 43126 Parma, Italy; valentinagiovanna.dellorto@unipr.it (V.D.); serafina.perrone@unipr.it (S.P.); 3 Neonatology Unit, Parma University Hospital, 43126 Parma, Italy

**Keywords:** neonatal pulmonary hypertension, persistent pulmonary hypertension of the newborn, neonatologist-performed echocardiography, functional echocardiography, right ventricular dysfunction, pulmonary vascular resistance

## Abstract

Neonatal pulmonary hypertension (PH) is a major cause of illness and death in newborns. Neonatologist-performed echocardiography (NPE) is increasingly used as a bedside tool to assess heart function, shunt patterns, and pulmonary blood flow in real time, helping clinicians better understand the severity and type of PH. This narrative review summarizes current evidence on the use of NPE in diagnosing, monitoring, and treating neonatal PH, drawing on clinical studies, guidelines, and expert recommendations. NPE provides key diagnostic and therapeutic information, including evaluation of ventricular function, estimation of pulmonary pressures, and assessment of shunt direction. Advanced measures—such as tricuspid annular plane systolic excursion (TAPSE), myocardial performance index, pulmonary artery acceleration time (PAAT), and deformation imaging—improve accuracy and help guide therapies like inhaled nitric oxide, milrinone, and sildenafil. NPE is also useful in chronic conditions such as bronchopulmonary dysplasia (BPD)- and congenital diaphragmatic hernia (CDH)-associated PH. Despite its clear clinical value, NPE use remains limited by variations in training, protocols, and resource availability. Standardized curricula, accreditation, and unified reporting practices are needed to ensure safe, consistent integration of NPE into neonatal care pathways.

## 1. Introduction

Neonatal pulmonary hypertension (PH) is a clinically significant condition characterized by elevated pulmonary vascular resistance (PVR), leading to right-to-left extrapulmonary shunting and severe hypoxemic respiratory failure (HRF) [1,2,3]. It reflects failure of the normal postnatal circulatory transition and affects both term and preterm infants. Three overlapping mechanisms underpin neonatal PH: maladaptation, involving sustained vasoconstriction triggered by perinatal stressors; underdevelopment, marked by reduced pulmonary vascular bed as seen in pulmonary hypoplasia or extreme prematurity; and maldevelopment, characterized by abnormal vascular remodeling and impaired vasoreactivity, typical of congenital diaphragmatic hernia (CDH) and severe bronchopulmonary dysplasia (BPD).

The most common presentation is Persistent Pulmonary Hypertension of the Newborn (PPHN), in which failure of the expected decline in PVR results in persistent fetal circulation. PPHN affects approximately 0.2% of live births and remains a major contributor to neonatal morbidity and mortality, particularly in term and late-preterm infants [4]. It is typically characterized by severe hypoxemia, right ventricular dysfunction, and right-to-left ductal or atrial shunting. In preterm infants, PH more often reflects pulmonary vascular immaturity and parenchymal lung disease, while late-onset PH—especially in evolving BPD—remains underrecognized despite clear associations with adverse outcomes [5,6].

Within this clinical landscape, neonatologist-performed echocardiography (NPE) has become a key bedside tool for real-time, non-invasive assessment of pulmonary and cardiovascular physiology. NPE assists in confirming PH after exclusion of congenital heart disease, assessing shunt dynamics, evaluating ventricular performance, and monitoring response to therapy. Its role is well established across conditions such as BPD, late-onset sepsis, and CDH [3,7,8,9,10,11] and supported by expert consensus guidelines promoting standardized use in neonatal PH [2,4,11,12]. Unlike point-of-care ultrasound (POCUS), which is suited to focused anatomical assessments, NPE enables comprehensive interrogation of cardiopulmonary physiology and is essential for guiding management in conditions such as PPHN, where small hemodynamic changes carry major therapeutic implications [13].

This narrative review synthesizes current evidence on the diagnostic, monitoring, and therapeutic applications of NPE in neonatal PH, with the aim of clarifying its evolving clinical role and outlining priorities for standardization and future practice.

## 2. Methods

### 2.1. Study Design

This work was conducted as a narrative review aimed at synthesizing contemporary evidence on the application of NPE and fECHO in the diagnosis, assessment, and management of neonatal PH. Given the heterogeneity of available data, the narrative format allowed for integration of clinical, physiological, and methodological perspectives drawn from diverse study designs, including observational studies, clinical trials, consensus guidelines, and expert reviews.

### 2.2. Literature Search Strategy

A comprehensive literature search was performed across multiple electronic databases, including PubMed/MEDLINE, Embase, Scopus, and the Cochrane Library, covering the period from January 1990 to December 2024. The last search was conducted in January 2025.

The search strategy combined Medical Subject Headings (MeSH) and free-text keywords related to neonatal pulmonary hypertension and echocardiography. The Boolean search string was: (“persistent pulmonary hypertension of the newborn” [Title/Abstract] OR “PPHN” [Title/Abstract] OR “neonatal pulmonary hypertension” [Title/Abstract] OR (“pulmonary hypertension” [Title/Abstract] AND neonate [Title/Abstract])) AND (“neonatologist-performed echocardiography” [Title/Abstract] OR “neonatal echocardiography” [Title/Abstract] OR “functional echocardiography” [Title/Abstract] OR “targeted neonatal echocardiography” [Title/Abstract] OR “NPE” [Title/Abstract] OR “fECHO” [Title/Abstract]) AND (“right ventricular function” [Title/Abstract] OR “pulmonary vascular resistance” [Title/Abstract] OR “PAAT” [Title/Abstract] OR “strain imaging” [Title/Abstract] OR “hemodynamics” [Title/Abstract]) NOT (“adult” [MeSH Terms] OR adult [Title/Abstract]). Reference lists of relevant articles and existing guidelines were manually screened to identify additional sources.

### 2.3. Eligibility Criteria

Studies were included if they met the following criteria:

1. Population: Term or preterm neonates (0–28 days) with suspected or confirmed pulmonary hypertension, or neonatal conditions where PH is a recognized component (e.g., BPD, CDH).
2. Intervention/Focus: Use of echocardiography for diagnosis, monitoring, or management of PH, including advanced modalities such as strain imaging or Doppler-derived indices.
3. Study Types: Observational studies, randomized trials, meta-analyses, expert consensus statements, guideline documents, and high-quality narrative reviews.
4. Language: English.

Exclusion criteria included:

- Studies not involving neonates (≥1 month of age).
- Case reports unless they offered unique insights relevant to physiology or methodology.
- Articles focused exclusively on congenital heart disease unrelated to PH physiology.
- Non-peer-reviewed opinion pieces, conference abstracts without published full text, and non-English publications.

### 2.4. Study Selection Process

All search results were screened by two independent reviewers (MF and EB) in two stages:

1. Title and Abstract Screening: Articles not meeting inclusion criteria were excluded.
2. Full-Text Review: The remaining studies were reviewed for final eligibility.

Any disagreements between reviewers were resolved through discussion and, if required, consultation with a third senior reviewer (LR). The final selection included seminal physiology papers, contemporary guidelines, and clinically impactful studies.

A total of 1240 records were identified through database searches and manual reference screening (illustrative number). After removal of 310 duplicates, 930 studies underwent title and abstract screening. Of these, 720 were excluded for not meeting inclusion criteria (e.g., non-neonatal population, absence of echocardiographic data, unrelated cardiopulmonary topic). The remaining 210 full-text articles were assessed for eligibility. Following full-text review, 142 studies were excluded, primarily due to lack of functional echocardiography data (*n* = 58), focus on congenital heart disease without PH relevance (*n* = 37), non-English language (*n* = 19), or insufficient methodological detail (*n* = 28). Ultimately, 68 studies were included in the qualitative synthesis.

Table 1 summarizes thematic domains and key findings from the included literature.

### 2.5. Data Extraction

Using a standardized extraction template, the following information was collected:

- Study design and year of publication
- Population characteristics and sample size
- Echocardiographic parameters studied (e.g., TRV, PAAT, TAPSE, RV strain)
- Clinical context (PPHN, BPD-associated PH, CDH-associated PH, preterm PH)
- Key findings related to diagnostic accuracy, prognostic value, and therapeutic implications
- Limitations cited by study authors

Special attention was given to parameters validated against invasive hemodynamics, studies assessing serial echocardiography, and trials evaluating echocardiography-guided interventions.

### 2.6. Synthesis of Evidence

Given the anticipated heterogeneity in methodology, populations, and outcome measures, data were synthesized qualitatively. Evidence was organized into thematic domains reflecting relevant clinical questions:

- Hemodynamic phenotyping of neonatal PH
- Role of NPE in acute PPHN
- Echocardiographic assessment in BPD-associated PH
- Assessment in CDH and pulmonary hypoplasia
- Advanced echocardiographic modalities
- NPE in therapeutic decision-making and monitoring
- Limitations, training variability, and global practice gaps

Consensus guideline recommendations were synthesized alongside primary research to provide a contemporary practice-focused perspective.

### 2.7. Quality Appraisal

Although formal risk-of-bias scoring was not performed due to the narrative nature of the review, studies were evaluated for methodological rigor using the following considerations:

- Appropriateness of study design for the clinical question
- Sample size and representativeness
- Clarity and reproducibility of echocardiographic methods
- Adjustment for confounders in observational studies
- Alignment with current consensus (e.g., NPE guidelines [3,11])

Guidelines and consensus statements were appraised for methodological transparency, multidisciplinary input, and clarity of recommendations.

### 2.8. Ethical Considerations

As this study involved a review of published literature without collection of new patient data, no ethics approval was required.

## 3. Hemodynamic Manifestations of Persistent Pulmonary Hypertension of the Newborn (PPHN)

PPHN is best understood as a dynamic and multifactorial circulatory disorder, rather than a purely pressure-mediated disease [14,15]. Although elevated PVR is the central abnormality and may independently cause HRF, disease severity is often compounded by a constellation of interacting hemodynamic disturbances (Table 2). These include right ventricular (RV) systolic dysfunction, interventricular septal displacement impairing left ventricular (LV) filling, and systemic hypoperfusion, together forming a self-perpetuating cycle of hypoxia, acidosis, and myocardial strain that limits responsiveness to pulmonary vasodilators. RV dysfunction, in particular, is increasingly recognized not as a secondary consequence but as a primary determinant of oxygenation and systemic output in PPHN.

From a hemodynamic standpoint, mean pulmonary artery pressure (mPAP) may be elevated due to one or more of the following:

- Increased PVR, driven by vasoconstriction and hypoxemia;
- Excessive pulmonary blood flow (Qp), as occurs with large left-to-right shunts or arteriovenous malformations;
- Elevated left atrial pressure (LAP), reflecting postcapillary pulmonary hypertension from LV diastolic dysfunction or volume overload.

These mechanisms give rise to three overlapping physiologic phenotypes of neonatal PH:

- Resistance-mediated PH, typically seen in parenchymal lung disease;
- Flow-mediated PH, resulting from excessive Qp and secondary vascular reactivity;
- Postcapillary PH, associated with LV dysfunction or pulmonary venous hypertension.

In early disease, the RV responds to increased afterload through homeometric adaptation, characterized by augmented contractility and concentric hypertrophy. With sustained pressure overload, however, the RV dilates, undergoes subendocardial ischemia, and progresses to systolic failure—a process described as heterotopic uncoupling. Septal flattening and leftward bowing further impair LV preload (ventricular interdependence), reducing systemic output and exacerbating hypoxemia in a closed cycle of deterioration [14,15,16]. These insights are consistent with classic findings by Skinner et al., who identified low LV output—not pulmonary pressure alone—as the most important early prognostic marker in PPHN [17].

In preterm infants, chronic PH is increasingly recognized, particularly in association with BPD and pulmonary vascular maldevelopment. While elevated PVR remains a hallmark, recent studies highlight additional contributors such as LV diastolic dysfunction, impaired myocardial compliance, and systemic hypertension, which can shift the hemodynamic profile toward postcapillary PH. Among prenatal contributors, intrauterine growth restriction (IUGR) and chronic fetal hypoxia are particularly important. Infants with placental insufficiency may develop late-onset acute PH and RV dysfunction following an initial period of postnatal stability, often temporally related to ductal closure and occurring in the absence of obvious parenchymal lung disease. These findings suggest that chronic intrauterine hypoxia produces structural and functional remodeling of both the pulmonary vasculature and the RV, increasing susceptibility to decompensation once the fetal shunts close [18].

Dhillon’s clinical classification distinguishes PH arising from parenchymal lung disease, idiopathic pulmonary vascular remodeling, and a reduced pulmonary vascular bed (e.g., in congenital diaphragmatic hernia or renal agenesis), aligning with the contemporary framework of maladaptation, maldevelopment, and underdevelopment [19]. This approach underscores the need for accurate hemodynamic phenotyping, as treatments beneficial in one phenotype (e.g., selective vasodilators in resistance-mediated PH) may be harmful in another (e.g., vasodilators in postcapillary PH without addressing LV dysfunction). Appropriate phenotyping is therefore critical to avoiding pulmonary edema, worsening oxygenation, or inadequate systemic perfusion.

## 4. Functional Echocardiography (ECHO) Assessment of Persistent Pulmonary Hypertension of the Newborn (PPHN)

FECHO has become integral to the bedside evaluation of neonates with suspected or confirmed pulmonary hypertension, providing immediate and non-invasive insights that extend well beyond diagnostic confirmation. It enables detailed characterization of the underlying pathophysiology of PPHN, supports accurate assessment of disease severity, guides therapeutic decision-making, and facilitates real-time monitoring of response to interventions. When performed by trained neonatologists (NPE), fECHO allows synthesis of structural and functional information within the clinical context of an infant whose hemodynamic status may change rapidly. Standardized guidelines issued by the European Special Interest Group on NPE promote a structured, reproducible, and developmentally appropriate approach to PH assessment in both term and preterm neonates [3]. A thorough examination begins with exclusion of congenital heart disease, since structural anomalies such as transposition of the great arteries, total anomalous pulmonary venous return, hypoplastic left heart syndrome, or coarctation of the aorta can mimic or aggravate PPHN and require entirely different management strategies. Once structural causes are ruled out, the examination proceeds to the estimation of pulmonary artery pressure using the tricuspid regurgitation (TR) jet when obtainable, or surrogate markers such as pulmonary regurgitation velocity, transductal shunt gradients, interventricular septal flattening, and the left ventricular systolic eccentricity index. Evaluation of shunt direction and magnitude across the ductus arteriosus and foramen ovale provides additional information regarding the severity of pulmonary vascular resistance, while an isolated right-to-left atrial shunt warrants urgent reassessment for possible total anomalous pulmonary venous return (TAPVR). Biventricular performance is then assessed using parameters including tricuspid annular plane systolic excursion (TAPSE), fractional area change, myocardial performance index, RV systolic/diastolic duration ratio, tissue Doppler imaging, and speckle-tracking echocardiography; left ventricular function is characterized through ejection fraction, stroke volume, cardiac output, and diastolic indices. In PPHN, right-to-left shunting and septal displacement often reduce LV preload, generating the appearance of LV dysfunction despite preserved intrinsic contractility.

Recent evidence has shifted practice away from a pressure-centric interpretation of echocardiographic findings toward a more integrated, physiology-based and phenotype-driven approach. Because RV systolic impairment can make pressure estimates unreliable, clinicians increasingly rely on a combination of RV performance metrics, shunt dynamics, and measures of systemic output [15]. Functional indices such as TAPSE, myocardial performance index (MPI), fractional area change (FAC), and left ventricular outflow tract velocity time integral (LVOT-VTI), together with ductal and atrial Doppler findings, offer a more comprehensive understanding of pulmonary vascular loading conditions [3,19]. When tricuspid regurgitation (TR) signals are absent, septal configuration and shunt directionality become particularly valuable [19]. Traditional clinical indicators—heart rate, blood pressure, capillary refill—are insufficient to capture the complexity of neonatal circulatory physiology [10], whereas fECHO enables direct assessment of preload, afterload, and output through parameters such as LVOT-VTI, TAPSE, inferior vena cava dynamics, and RV fractional area change [5]. These detailed evaluations have been shown to alter clinical management in up to 60% of cases, underscoring their importance in contemporary neonatal intensive care [9]. Importantly, fECHO is not a static diagnostic tool but a dynamic monitoring strategy. Serial assessments allow early detection of changes in ventricular performance, shunt behavior, and pulmonary pressures, and provide critical guidance during titration of vasodilators, inotropes, and ventilatory support. By adopting a comprehensive, physiology-based echocardiographic framework, clinicians are better equipped to stratify risk, tailor interventions, and improve outcomes for infants affected by this highly dynamic and life-threatening condition.

Table 3 summarizes key echocardiographic parameters in PPHN.

## 5. Advanced Echocardiographic Parameters and Cardiac Catheterization

Advances in functional echocardiography have markedly expanded the diagnostic and monitoring capabilities available for neonatal pulmonary hypertension, allowing for a more nuanced, real-time assessment of right and left ventricular performance, pulmonary vascular resistance, and overall hemodynamic state. Modern techniques incorporate multiple quantitative parameters—many of which correlate closely with invasive measurements obtained through right heart catheterization. Although cardiac catheterization remains the gold standard for differentiating pre- and postcapillary PH, identifying pulmonary vein stenosis or anomalous venous return, and evaluating infants who fail to respond to optimized medical therapy, its application is limited by procedural risk and instability in critically ill neonates. In these circumstances, advanced echocardiographic markers provide a valuable and often indispensable surrogate for invasive assessment [20]. The integration of these modalities enables clinicians to refine diagnosis, stratify disease severity, and guide decisions surrounding escalation to advanced therapies, including targeted pharmacologic interventions or extracorporeal membrane oxygenation (ECMO). Table 4 shows advanced echocardiographic parameters and cardiac catheterization in neonatal PH.

### 5.1. Right Ventricular Function and Pressure Overload

Accurate characterization of RV systolic function and pressure load is central to the evaluation of neonatal PH, as RV performance is closely linked with outcomes in PPHN. Several echocardiographic indices have been validated in neonatal populations:

- TAPSE: A robust measure of longitudinal RV function. TAPSE values <4 mm are associated with increased risk of ECMO or mortality and have strong reproducibility, although influenced by loading conditions and insonation angle [10,21].
- Fractional Area Change (FAC): Derived from apical four-chamber views; FAC < 20% suggests impaired RV systolic function. Three-dimensional speckle-tracking echocardiography (3D-STE) offers enhanced sensitivity compared with 2D measurements [2,22].
- RV Systolic/Diastolic Duration Ratio (S/D Ratio): Obtained from Doppler interrogation of the TR jet. Ratios > 1.3 indicate abnormal RV performance and correlate with adverse outcomes [23].
- Myocardial Performance Index (MPI or Tei Index): Combines systolic and diastolic intervals to reflect global RV function. Thresholds > 0.55 by tissue Doppler or >0.40 by pulsed-wave Doppler suggest significant dysfunction [20].
- Left Ventricular Systolic Eccentricity Index (LV-sEI): Assessed in parasternal short-axis views; values > 1.0 reflect interventricular septal flattening due to elevated RV pressure load [15,24].

Together, these parameters offer complementary perspectives on RV performance and afterload, supporting early risk stratification and tailored therapeutic interventions.

### 5.2. Estimation of Pulmonary Vascular Resistance

When cardiac catheterization is not feasible, several non-invasive echocardiographic surrogates assist in estimating PVR:

- Pulmonary Artery Acceleration Time (PAAT): Shortened PAAT (<90 ms) indicates elevated PVR, while values < 40 ms strongly suggest severe PH. PAAT correlates inversely with both mean and systolic pulmonary artery pressures [24,25,26].
- PAAT/Right Ventricular Ejection Time (PAAT/RVET) Ratio: A ratio < 0.23 is associated with significantly increased pulmonary pressures in neonates with PH [20].
- Tricuspid Regurgitation Velocity (TRV): When combined with RV outflow tract velocity-time integral (RVOT VTI), the TRV/VTI ratio may approximate PVR, though additional neonatal validation is required.
- Pulmonary Artery Compliance: Assessed by systolic–diastolic diameter variation; decreased compliance reflects increased vascular stiffness but remains technically challenging in neonates.

Early work by Kadivar et al. validated PAAT and PAAT/RVET as reliable non-invasive surrogates for elevated PVR in neonates with PPHN, demonstrating a strong inverse correlation with estimated pulmonary pressures [12]. Despite their utility, these indices are sensitive to technical factors such as image quality, respiratory mechanics, heart rate variability, and probe angle. High-quality imaging and careful interpretation are therefore essential in neonatal populations [10,20].

### 5.3. Strain Imaging and Deformation Analysis

Deformation imaging enables the early detection of RV dysfunction, often preceding abnormalities in conventional indices such as TAPSE or FAC [1,20]. Several modalities contribute to a detailed understanding of myocardial mechanics:

- Speckle-Tracking Echocardiography (STE): Provides quantitative assessment of global longitudinal strain (GLS). RV GLS > –9% predicts need for ECMO, and reduced GLS correlates with disease severity and improves with effective therapy [21].
- Tissue Doppler Imaging (TDI): Evaluates myocardial velocities at the tricuspid annulus (s′, e′, a′). Reduced e′ velocities reflect impaired diastolic relaxation and predict poorer outcomes.
- Right Atrial (RA) Strain: Offers characterization of reservoir, conduit, and pump function. Abnormal RA strain is associated with RV dysfunction and adverse clinical outcomes [21,22].

Combined interpretation of RV GLS and RA strain enhances early recognition of right heart stress and supports timely therapeutic adjustments. The integration of STE and TDI thus provides a sensitive, longitudinal assessment of RV function, refining risk stratification and management decisions in neonatal PH.

### 5.4. Left Ventricular Function

Assessment of LV performance is essential, particularly for identifying postcapillary contributors to PH and understanding ventricular interdependence [4,20]. Key parameters include:

- LV Fractional Shortening (FS): Typically preserved in PPHN unless septal flattening impairs mechanical efficiency.
- Pressure-Strain Loop (PSL): Provides insight into myocardial work, including global constructive work (GCW), wasted work (GWW), efficiency (GWE), and index (GWI). PSL metrics correlate with outcomes in adult PH and show promise in neonatal populations [20].
- LV Tei Index: A global measure of LV performance; in some neonatal studies, it surpasses the RV Tei Index in prognostic value.

Although many neonatal datasets are limited by small sample sizes, foundational adult studies—such as those by Fukuda et al.—demonstrated that RV free-wall strain closely correlates with invasively measured mPAP and PVR [27]. These findings have informed the adaptation of advanced deformation imaging for neonatal cardiopulmonary evaluation.

Taken together, advanced echocardiographic techniques, when interpreted within the broader clinical context, serve as a cornerstone of contemporary neonatal PH management. Functional echocardiography should be viewed as an extension of the neonatal physical examination—dynamic, context-sensitive, and indispensable for individualized, physiology-driven care in critically ill infants [10].

## 6. Utility of NPE in the Management of Persistent Pulmonary Hypertension of the Newborn (PPHN)

Effective management of PPHN requires careful identification of the dominant hemodynamic disturbances and the use of targeted, physiology-based interventions. Jain and McNamara proposed a structured framework in which optimization of lung function (oxygenation, surfactant therapy, and ventilatory support), correction of acidosis, and stabilization of the circulation form the foundation of care, followed by selective use of pulmonary vasodilators guided by functional assessment [15].

### 6.1. Echo-Guided Inhaled Nitric Oxide Therapy

NPE plays a central role in this approach by enabling real-time evaluation of PVR, RV function, and LV preload—all critical determinants of response to inhaled nitric oxide (iNO) and other vasodilators.

The clinical relevance of echo-guided decision-making is highlighted by national data from Japan, where Shiraishi et al. reported that 100% of surveyed NICUs used echocardiography to guide initiation of iNO, including in extremely preterm infants (<28 weeks gestation) [28]. Most units commenced iNO at ≤10 ppm and monitored response via differential SpO_2_ and oxygenation index (OI), initiating weaning once OI improved (<10) or saturations normalized. Serial echocardiograms were typically performed every 4–8 h to evaluate RV performance, shunt direction, and systemic output, guiding individualized titration and withdrawal over 12–24 h. This real-world experience demonstrates the feasibility and utility of continuous, echo-directed iNO therapy even in physiologically fragile infants.

Broad regional surveys reinforce the growing reliance on NPE. In Australia and New Zealand, the majority of NICUs report using NPE to guide iNO therapy in preterm infants despite the absence of standardized protocols [29]. When integrated into routine care, NPE becomes an extension of the clinical examination, allowing clinicians to tailor interventions to the infant’s evolving hemodynamic profile. Key studies illustrate the importance of serial evaluations: low LV output (<100 mL/kg/min) and low stroke volume index (SVI < 1 mL/kg) are strongly associated with mortality, even in infants with preserved LV systolic function [17]. In contrast, initial estimates of pulmonary pressure or early shunt patterns do not reliably predict outcomes. Breinig et al. similarly demonstrated that persistence of right-to-left shunting across the ductus arteriosus or foramen ovale on a repeat echocardiogram—rather than on the initial study—was independently associated with mortality before day 28, underscoring the prognostic value of dynamic, repeated assessments [30].

### 6.2. Adjunctive Pharmacological Strategies

Dhillon’s phenotype-based management algorithm further supports integrating NPE into therapeutic decision-making. iNO remains first-line therapy in term infants with PPHN and has strong evidence for improving oxygenation and reducing ECMO use [19]. For inadequate responders, adjunctive agents such as sildenafil or milrinone may be added, with the latter particularly beneficial in the presence of ventricular dysfunction because of its combined inotropic and lusitropic effects. Prostaglandin E1 (PGE_1_) is increasingly recognized as a potentially valuable adjunct beyond its traditional role in duct-dependent congenital heart disease, particularly in infants with severe PH and RV compromise.

### 6.3. Predictors of Escalation and Extracorporeal Membrane Oxygenation

Malowitz et al. identified several echocardiographic predictors of progression to ECMO or cardiovascular death, including TAPSE < 4 mm, impaired RV strain (GLPS ≥ –9%), and predominantly right-to-left ductal shunting. TAPSE demonstrated independent predictive value even after accounting for oxygenation index (OI > 20), emphasizing the importance of integrating functional markers with oxygenation metrics when escalating therapy [21].

When maximal medical therapy fails and OI exceeds 40, ECMO should be considered in the absence of contraindications. In such cases, ongoing echocardiographic monitoring remains essential to assess reversibility, guide escalation, and determine the optimal timing for cannulation. Jain and McNamara highlight the limitations of relying solely on clinical surrogates—blood pressure, urine output, capillary refill—which frequently fail to capture the complexity of neonatal circulatory physiology. Instead, they advocate for routine use of real-time NPE to precisely delineate the predominant hemodynamic disturbance and support targeted therapy [15]. Adjunctive medications—including sildenafil, milrinone, vasopressin, prostacyclins, magnesium sulfate, and bosentan—can then be selected based on detailed echocardiographic characterization, with the overarching goal of optimizing the PVR:SVR ratio and improving both shunt dynamics and systemic perfusion [31].

### 6.4. Global Practice Variability

Supporting the RV is another central tenet of PPHN management. RV dysfunction arising from ischemia, inflammation, or excessive afterload compounds hypoxemia and systemic instability. Therapeutic strategies must therefore preserve RV preload, avoid excessive intrathoracic pressures, and enhance contractility through agents such as milrinone or dobutamine. The utility of NPE in differentiating forms of decompensation is well illustrated by Danhaive et al., who described a cohort of extremely low birthweight, growth-restricted infants developing acute RV dysfunction following ductal closure. NPE revealed markedly reduced RV ejection fraction (median 28%), guiding a combination of ventilatory adjustments, inotropes, and milrinone; improvements in echocardiographic markers paralleled clinical recovery, underscoring the value of physiologic phenotyping in distinguishing PH-related deterioration from alternative etiologies such as sepsis [18].

Several authors have proposed structured frameworks that synthesize echocardiographic indices into clinically actionable pathways. Bhattacharya et al. organized measurements into three domains: indirect markers of RV afterload (septal flattening, eccentricity index), estimators of pulmonary hemodynamics (TRV, PAAT), and measures of RV performance (TAPSE, FAC, MPI, RV strain), demonstrating how quantitative indices can identify early physiologic abnormalities before overt clinical signs appear [22]. More et al. further integrated echocardiographic findings into a stepwise bedside algorithm that begins with exclusion of congenital heart disease and progresses through systematic evaluation of pulmonary pressures, shunting patterns, and ventricular function [4]. Shunt directionality across the PDA and PFO provides immediate insight into disease severity and therapeutic needs: bidirectional or right-to-left shunting suggests suprasystemic PH requiring aggressive vasodilation and inotropic support, while left-to-right shunting reflects milder disease. Mixed patterns often indicate concurrent LV dysfunction, warranting caution with vasodilators and favoring milrinone. These authors emphasize that no single parameter—such as TR velocity—should be interpreted in isolation, and that serial, integrated assessment is essential for safe, precise management.

Importantly, the growing sophistication of NPE-guided management must be considered within a global context. Nakwan provides a perspective from low-resource settings, where functional echocardiography, iNO, ECMO, and pediatric cardiology support may be unavailable [32]. In such environments, clinicians rely on clinical markers such as pre-/post-ductal SpO_2_ gradients and hyperoxia or hyperoxia-hyperventilation tests to identify PH. Management follows a structured care-bundle approach emphasizing targeted oxygen therapy, hemodynamic stabilization, sedation, and the use of accessible vasodilators such as sildenafil, iloprost, milrinone, magnesium sulfate, and bosentan. While echo-based monitoring is not feasible, the underlying principles of physiology-guided care remain applicable, reinforcing the value of disseminating echocardiographic training and infrastructure globally to promote equity in neonatal PH management.

**Figure 1:**
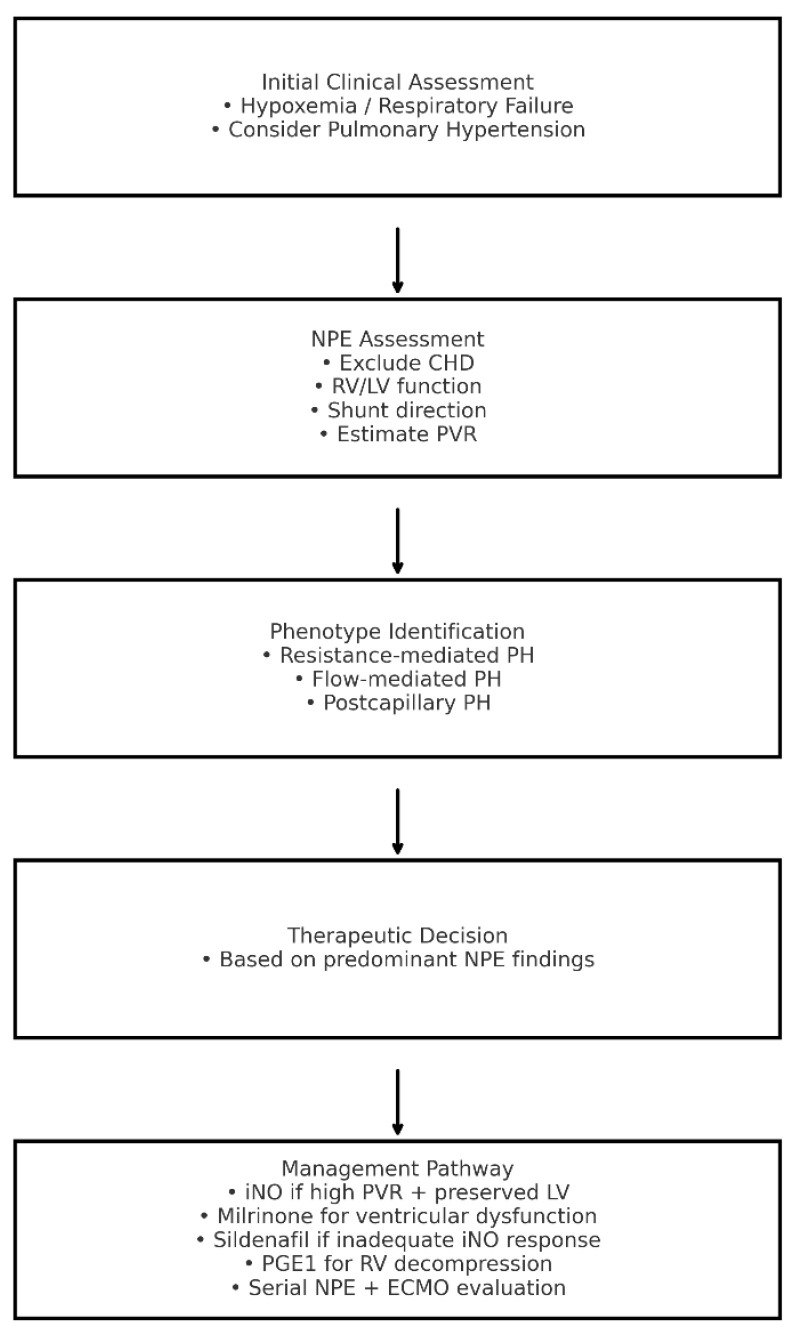
Algorithm for the Assessment and Management of Neonatal Pulmonary Hypertension Integrating Neonatologist-Performed Echocardiography Findings. CHD: congenital heart disease; ECMO: extracorporeal membrane oxygenation; iNO: inhaled nitric oxide; LV: left ventricle; NPE: neonatologist-performed echocardiography; RV: right ventricle; PGE1: prostaglandin E1; PH: pulmonary hypertension; PVR: pulmonary vascular resistance.

In summary, NPE has evolved from a diagnostic adjunct into a cornerstone of personalized, physiology-driven management in neonatal PH. When performed and interpreted by skilled neonatologists, it enables real-time hemodynamic assessment, supports tailored therapy, and facilitates early recognition of deterioration (Figure 1). This integrated, dynamic approach represents a paradigm shift in PPHN management and aligns closely with the principles of precision neonatal medicine.

## 7. Specific Considerations

### 7.1. BPD-Associated Pulmonary Hypertension

BPD-associated pulmonary hypertension is a major contributor to morbidity and mortality in extremely preterm infants. Its pathogenesis is multifactorial, driven by disrupted alveolar and vascular development, chronic inflammation, oxygen toxicity, and mechanical ventilation. Characteristic pathological features—including alveolar simplification, reduced intra-acinar vessel density, and abnormal muscularization of distal pulmonary arteries—lead to elevated PVR, impaired gas exchange, and increased RV afterload. Clinical management focuses on meticulous oxygen titration to prevent hypoxic pulmonary vasoconstriction, optimization of lung growth through nutrition and avoidance of infection, and cautious use of pulmonary vasodilators. Although agents such as sildenafil show promise in animal models and small human cohorts, several authors caution against indiscriminate use because of the risk of ventilation–perfusion mismatch, emphasizing the importance of individualized, echo-guided therapy [6,19].

Conventional echocardiographic markers—interventricular septal flattening, RV hypertrophy, and tricuspid regurgitation-derived systolic pressure estimates—are commonly applied but often limited in preterm infants due to poor acoustic windows and low signal quality. To enhance early detection, Patel et al. prospectively evaluated 239 infants < 29 weeks gestation, establishing maturational reference patterns for PAAT and the PAAT/RV ejection time (RVET) ratio as non-invasive surrogates of RV afterload. In healthy infants, both indices rose with gestational and postnatal age, reflecting the expected postnatal decline in PVR. Infants who later developed BPD or PH showed persistently reduced PAAT values and flattened maturational trajectories extending through the first year of corrected age. A PAAT < 47 ms and PAAT/RVET < 0.28 at 32 weeks postmenstrual age predicted PH at 36 weeks with >90% sensitivity and specificity [33]. Importantly, these indices were obtainable far more consistently than tricuspid regurgitation velocities, which were measurable in fewer than 10% of studies.

Thus, PAAT and PAAT/RVET have emerged as practical and reliable tools for early identification, risk stratification, and longitudinal follow-up in infants at risk for BPD-associated PH. Savoia et al. further demonstrated their feasibility across inpatient and outpatient settings, showing that subtle deviations in PAAT trajectories identified infants who later developed clinically mild, self-resolving PH [6]. Combined with traditional evaluation, these parameters provide a sensitive framework for detecting evolving pulmonary vascular disease before overt decompensation.

Table 5 summarizes echo markers of BPD-associated PH.

### 7.2. Congenital Diaphragmatic Hernia (CDH)-Associated Pulmonary Hypertension

Pulmonary hypertension in CDH arises from a combination of pulmonary hypoplasia and abnormal pulmonary vascular remodeling, making it one of the most severe neonatal PH phenotypes. These infants typically exhibit markedly elevated PVR, diminished responsiveness to vasodilator therapy, and a high risk of adverse outcomes. Although iNO is widely used, randomized trials have not demonstrated consistent benefit in CDH [19]. Nonetheless, iNO may still be reasonable as a first-line agent when its use is accompanied by serial echocardiographic evaluation to assess responsiveness and avoid unnecessary exposure.

Echocardiography remains indispensable in CDH for confirming PH, monitoring disease progression, and informing the timing of surgical repair. Assessments of RV function, systemic output, and shunt dynamics—particularly the direction of ductal and atrial flow—guide perioperative decision-making and help distinguish infants likely to benefit from vasodilators, inotropes, or delayed surgical correction. In this population, NPE serves not only as a diagnostic tool but also as a key determinant of therapeutic strategy throughout stabilization, operative planning, and postoperative recovery.

Table 6 summarizes echo findings and clinical implication in CDH-associated PH.

### 7.3. Adjunct and Emerging Therapies in Persistent Pulmonary Hypertension of the Newborn (PPHN)

For infants with PPHN who do not respond adequately to first-line therapies such as iNO and surfactant, adjunct medications can play a decisive role in restoring hemodynamic stability. Milrinone, a phosphodiesterase-3 inhibitor, provides balanced pulmonary vasodilation and inotropic support by increasing intracellular cAMP. It is particularly helpful in infants with LV dysfunction or pulmonary venous hypertension, where iNO alone may worsen pulmonary edema. Because of the risk of systemic hypotension, milrinone should be initiated without a loading dose, and preload should be optimized; low-dose epinephrine may be required to maintain perfusion [5].

Sildenafil, a phosphodiesterase-5 inhibitor, enhances cGMP-mediated vasodilation and is useful when iNO is unavailable, ineffective, or when chronic vasodilator therapy is required. An echocardiographic evaluation is recommended prior to initiation to exclude significant LV dysfunction, which may predispose to systemic hypotension. Prostaglandin E1 (PGE1) represents another valuable adjunct in infants with a constricting ductus arteriosus and RV failure, as maintaining ductal patency can create a “pop-off” mechanism that reduces RV afterload via right-to-left shunting.

Emerging therapies are under investigation for infants with refractory PH (Table 7). Soluble guanylate cyclase (sGC) activators, Rho-kinase inhibitors, and antioxidant agents such as superoxide dismutase aim to correct abnormalities in NO signaling, redox imbalance, and vascular remodeling. Although promising, these agents require further study to define safety, dosing, and clinical utility before incorporation into standard practice. As understanding of the molecular drivers of PPHN advances—including dysregulation of the NO–cGMP axis, prostacyclin–cAMP signaling, and endothelin pathways—future therapies may allow more precise tailoring of treatment to individual hemodynamic phenotypes.

### 7.4. Transitional and Long-Term Considerations in Neonatal Pulmonary Hypertension (PPHN)

Although clinical management of neonatal PH often centers on acute stabilization, growing evidence underscores the need for structured long-term follow-up, particularly in infants with persistent, evolving, or high-risk phenotypes such as BPD- or CDH-associated PH. Increasingly, neonatal PH is recognized not as an isolated perinatal event but as the early manifestation of a chronic pulmonary vascular disorder, especially in preterm infants and those with ongoing lung disease [2]. This shift warrants a lifespan-oriented model of care that bridges neonatology, pediatric cardiology, and pulmonology.

A major challenge remains the absence of standardized transition protocols for infants discharged after PH treatment. Many do not receive ongoing monitoring for recurrence, progression, or long-term complications, risking delayed identification of fixed pulmonary vascular disease. Echocardiographic findings—including persistent RV dysfunction, abnormal septal motion, or limited oxygenation response—should be incorporated into discharge planning and flagged for early referral to specialized PH centers [2]. Integrating echocardiographic data into post-discharge clinics and PH registries will help ensure continuity of care.

Multidisciplinary follow-up is essential for optimizing outcomes, particularly as symptoms of chronic PH may be subtle in infancy and early childhood. The evolving understanding of neonatal PH necessitates a transition from reactive management to proactive, integrated surveillance. Neonatologists are uniquely positioned to lead this shift by establishing structured pathways that ensure infants with early PH—especially those born extremely preterm or with CDH—receive comprehensive long-term monitoring and timely access to specialized care.

**Figure 2:**
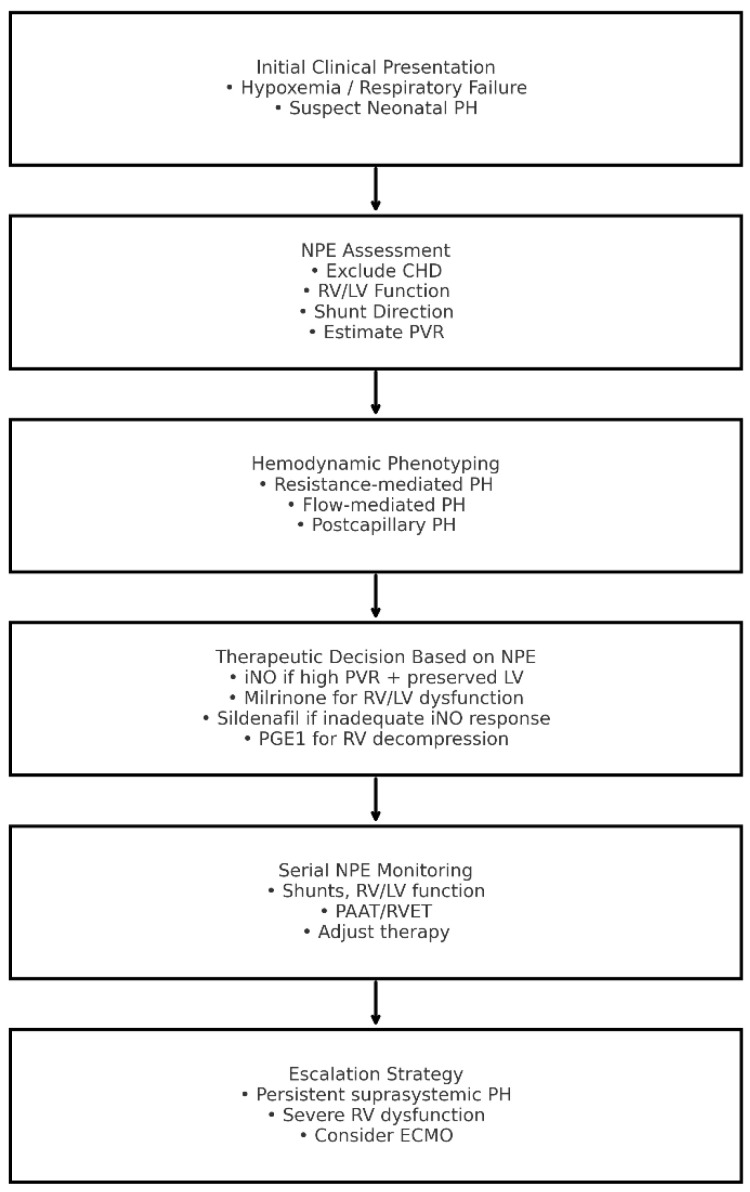
Neonatologist-Performed Echocardiography-Integrated Clinical Pathway for Neonatal Pulmonary Hypertension. CHD: congenital heart disease; ECMO: extracorporeal membrane oxygenation; iNO: inhaled nitric oxide; LV: left ventricle; NPE: neonatologist-performed echocardiography; PAAT: pulmonary artery acceleration time; PH: pulmonary hypertension; PGE1: prostaglandin E1; PVR: pulmonary vascular resistance; RV: right ventricle; RVET: right ventricular ejection time.

Figure 2 summarizes NPE-integrated clinical pathway for neonatal PH.

## 8. Pitfalls

Despite the expanding role of f-ECHO in neonatal hemodynamic assessment, its clinical implementation remains challenged by significant practical and methodological limitations. A nationwide Italian survey revealed that although f-ECHO is routinely used in nearly all centers, substantial variability persists in clinical practice, training pathways, and procedural standardization [7]. One of the most pressing concerns is the lack of institutional protocols: nearly one-third of surveyed units lacked formal guidelines for echocardiographic management of key conditions such as persistent pulmonary hypertension of the newborn (PPHN) or patent ductus arteriosus. This absence of structured frameworks contributes to heterogeneity in diagnostic assessment and therapeutic decision-making, ultimately compromising consistency and potentially affecting outcomes.

Furthermore, while estimation of pulmonary artery pressure is commonly attempted, more advanced indices of RV performance—such as TAPSE, FAC, and MPI—remain underutilized. The omission of these parameters limits the ability to accurately characterize RV function and appropriately phenotype pulmonary hypertension. Training variability further exacerbates this issue. Many neonatologists acquire echocardiography skills through informal or non-standardized training experiences, and relatively few complete accredited programs, raising concerns regarding diagnostic reliability—particularly in distinguishing structural cardiac anomalies from functional disturbances in neonatal PH [8].

The need for harmonized training and accreditation is highlighted across multiple regions. A binational survey of neonatologists in Canada and Australasia identified substantial variation in PPHN management strategies, including differences in iNO initiation thresholds, choice of adjunct vasodilators, and availability of neonatologist-performed echocardiography. Notably, formal training remained uncommon despite widespread reliance on f-ECHO for clinical decision-making [28]. Technical limitations compound these challenges: the lack of ECG integration, inconsistent digital archiving, and non-standardized reporting formats reduce study reproducibility, impede longitudinal follow-up, and limit opportunities for quality assurance.

Methodological limitations also affect the reliability of echocardiographic indices used to estimate PVR. When tricuspid regurgitant velocity is unobtainable—a frequent occurrence in premature or critically ill neonates—clinicians often rely on surrogate markers such as PAAT or the PAAT/RVET ratio. While Kadivar et al. established a robust inverse correlation between PAAT and estimated pulmonary pressures in term infants with PPHN [12], subsequent studies have demonstrated that these measurements are sensitive to ventilatory settings, probe angle, heart rate, and operator expertise, underscoring the need for standardized acquisition protocols.

Although collaboration with pediatric cardiologists is widely practiced, real-time cardiology support is often unavailable during acute deterioration. As a result, neonatologists may be solely responsible for image acquisition, interpretation, and critical management decisions during high-stakes situations. This reality amplifies the need for comprehensive training, governance structures, and shared protocols to ensure safe and physiologically accurate use of f-ECHO across diverse NICU settings.

In summary, while functional echocardiography has become an indispensable component of modern neonatal intensive care, its safe and effective application requires more than technical competency. Standardized protocols, structured accreditation pathways, rigorous quality assurance systems, and strengthened interdisciplinary collaboration are essential to minimize variability, improve diagnostic precision, and enhance patient outcomes. Without these safeguards, the risk of misclassification, mismanagement, and missed diagnoses remains unacceptably high—particularly in neonates with pulmonary hypertension, a group for whom diagnostic and therapeutic decisions are especially time-critical.

## 9. Conclusions

NPE has fundamentally reshaped the evaluation and management of neonatal pulmonary hypertension by enabling precise, physiology-based assessment at the bedside. Its ability to characterize pulmonary pressures, shunt direction, and ventricular performance in real time has replaced empiric decision-making with targeted, responsive care. Across acute PPHN, BPD-associated PH, and CDH-associated PH, NPE now serves as a core tool for stabilization, serial monitoring, and prognostication, with emerging modalities—such as strain imaging and PAAT-derived indices—further enhancing early detection and phenotyping.

Despite this progress, major challenges persist. Variability in training, limited access to accredited pathways, and inconsistency in measurement and reporting standards continue to hinder widespread, reliable adoption. Resource limitations, particularly in centers without specialized cardiology support, compound these barriers and amplify global disparities in care. Addressing these gaps requires coordinated efforts to develop structured curricula, universal competency benchmarks, and harmonized clinical protocols that ensure safe, reproducible application across diverse neonatal settings.

Looking ahead, the role of NPE must extend beyond the acute phase and into long-term follow-up, especially as neonatal PH increasingly evolves into a chronic condition among preterm infants with ongoing lung disease. Integration of functional echocardiographic data into multidisciplinary follow-up programs, PH registries, and longitudinal surveillance frameworks will be essential to anticipating late cardiopulmonary complications and guiding individualized, lifespan-oriented care.

To support consistency and equity in clinical practice, we propose that all neonatologists be familiar with a minimum essential set of echocardiographic and clinical parameters—specifically, ventricular function (TAPSE or FAC), shunt direction across the ductus and foramen ovale, qualitative estimates of pulmonary vascular resistance (PAAT or septal configuration), and basic systemic hemodynamic markers (LV function and cardiac output)—as these elements provide the fundamental orientation required for initial diagnostic classification and therapy planning. Advancing the field will depend on broader training, standardized practice, and sustained investment in NPE infrastructure to ensure that every infant with pulmonary hypertension receives timely, physiology-guided, and high-quality care.

## Figures and Tables

**Table 1:** Thematic Domains and Key Findings from the Included Literature.

Thematic Domain	Number of Included Studies	Key Findings
Hemodynamic Phenotyping of Neonatal PH	12	PH results from overlapping mechanisms (maladaptation, maldevelopment, underdevelopment); RV dysfunction and septal shift strongly influence severity.
Role of NPE in Acute PPHN	18	NPE refines diagnosis, evaluates shunt direction, and guides therapies such as inhaled nitric oxide and vasodilators.
Echocardiographic Assessment in BPD-associated PH	10	PAAT and PAAT/RVET are reliable early markers; abnormal trajectories predict PH and adverse outcomes.
Assessment in CDH-associated PH	8	Severe RV dysfunction and fixed R→L shunting predict poor vasodilator response; NPE guides timing of repair.
Advanced Echocardiographic Modalities	9	Strain imaging and myocardial work indices detect early dysfunction and correlate with PH severity.
NPE-Guided Therapeutic Decision-Making	15	NPE alters management in a large proportion of cases; supports individualized titration of vasodilators/inotropes and ECMO decisions.
Training and Standardization Gaps	6	Substantial global variability in training, protocols, and reporting standards; need for harmonized curricula.

BPD: Bronchopulmonary Dysplasia; CDH: Congenital Diaphragmatic Hernia; ECMO: Extracorporeal Membrane Oxygenation; NPE: Neonatologist-Performed Echocardiography; PAAT: Pulmonary Artery Acceleration Time; PH: Pulmonary Hypertension; PPHN: Persistent Pulmonary Hypertension of the Newborn; RV: Right Ventricle; RVET: Right Ventricular Ejection Time.

**Table 2:** Mechanistic Classification of Neonatal Pulmonary Hypertension.

Mechanism	Key Pathophysiology	Common Clinical Contexts	Echocardiographic Features	Therapeutic Implications
Maladaptation	Sustained vasoconstriction; impaired transition	Meconium aspiration, sepsis, asphyxia	↑PVR, right-to-left shunting	Responds to selective pulmonary vasodilators (e.g., iNO); optimize lung recruitment; treat underlying triggers (infection, hypoxia).
Maldevelopment	Abnormal vascular remodeling	CDH, severe BPD	RV hypertrophy, septal flattening	Often limited response to iNO; may require multimodal therapy (sildenafil, milrinone); careful fluid and ventilatory management.
Underdevelopment	Reduced vascular bed	Pulmonary hypoplasia, extreme prematurity	Low PAAT, high RVET	Focus on lung-protective ventilation and gradual oxygenation targets; limited vasodilator responsiveness; support cardiac output.

BPD: Bronchopulmonary Dysplasia; CDH: Congenital Diaphragmatic Hernia; iNO: inhaled nitric oxide; PAAT: Pulmonary Artery Acceleration Time; PVR: Pulmonary Vascular Resistance; RV: Right Ventricular; RVET: Right Ventricular Ejection Time; ↑, Increase.

**Table 3:** Key Echocardiographic Parameters in Persistent Pulmonary Hypertension of the Newborn (PPHN).

Parameter	Interpretation	Normal Range	Pathologic Threshold
TAPSE	RV systolic function	>6 mm	<4 mm
FAC	RV area change	>30%	<20%
MPI (Tei)	Global RV function	<0.40 PW; <0.55 TDI	>thresholds
LV-sEI	Septal position, RV load	≈1.0	>1.0
PAAT	PVR surrogate	90–120 ms	<40–50 ms
PAAT/RVET	Adjusted PVR	>0.25	<0.23

FAC: Fractional Area Change; LV-sEI: Left Ventricular Systolic Eccentricity Index; MPI (Tei): Myocardial Performance Index (Tei Index); PAAT: Pulmonary Artery Acceleration Time; PAAT/RVET: Pulmonary Artery Acceleration Time to Right Ventricular Ejection Time Ratio; PVR: Pulmonary Vascular Resistance; PW: Pulsed-Wave (Doppler); RV: Right Ventricular; TAPSE: Tricuspid Annular Plane Systolic Excursion; TDI: Tissue Doppler Imaging.

**Table 4:** Advanced Echocardiographic Parameters and Cardiac Catheterization in Neonatal Pulmonary Hypertension.

Parameter/Modality	What It Measures	Typical Thresholds/Abnormal Findings	Clinical Significance
TAPSE (Tricuspid Annular Plane Systolic Excursion)	Longitudinal RV systolic function	<4 mm suggests significant RV dysfunction	Predicts severity and risk of ECMO or mortality; guides need for inotropic support.
RV Fractional Area Change (FAC)	Global RV systolic performance	<20% indicates impaired RV function	Strong indicator of RV failure; helps assess response to vasodilators and inotropes.
Myocardial Performance Index (MPI/Tei Index)	Combined RV systolic and diastolic performance	>0.40 (PW) or >0.55 (TDI) is abnormal	Early marker of global RV dysfunction; useful when TR signal is absent.
PAAT (Pulmonary Artery Acceleration Time)	Surrogate of PVR	<40–50 ms suggests severe PH	Reliable non-invasive estimator of PVR; tracks disease progression, especially in preterm infants.
PAAT/RVET Ratio	Adjusts PAAT for heart rate	<0.23 indicates very high PVR	Helpful when PAAT is shortened by tachycardia; improves accuracy in neonates.
TRV (Tricuspid Regurgitation Velocity)	RV systolic pressure estimate	Elevated Doppler gradient; may be unobtainable	When measurable, approximates RV pressure; limited by poor signal quality in neonates.
Septal Configuration/Eccentricity Index (sEI)	RV pressure load and ventricular interdependence	sEI > 1.0 indicates RV pressure overload	Useful when TRV is absent; reflects severity of PVR elevation and LV preload reduction.
RV Systolic/Diastolic Duration Ratio (S/D Ratio)	Impact of afterload on RV contraction	>1.3 suggests impaired RV performance	Correlates with disease severity and outcomes; highly reproducible.
Speckle-Tracking Echocardiography (RV GLS)	RV deformation/contractility	RV GLS > –9% severely abnormal	Detects early dysfunction before TAPSE or FAC decline; strong prognostic marker.
Right Atrial Strain	RA reservoir and conduit function	Reduced RA strain indicates RV pressure overload	Complements RV strain; associates with poor outcomes and impaired RV filling.
LV Pressure-Strain Loop (Myocardial Work Indices)	LV systolic performance accounting for afterload	Altered GCW/GWE in PH	Helps detect postcapillary or mixed PH; useful for distinguishing LV vs. RV-driven disease.
Cardiac Catheterization	Direct measurement of PA pressure, PVR, pulmonary venous anatomy	Elevated mPAP, PVR, or evidence of PV obstruction	Gold standard; essential when PH is refractory, anatomy is unclear, or pre/postcapillary differentiation is needed; limited by procedural risk in neonates.

GCW: global constructive work; GLS: global longitudinal strain; GWE: global work efficiency; mPAP: mean pulmonary artery pressure; LV: left ventricle; PVR: pulmonary vascular resistance; PAAT: pulmonary artery acceleration time; RV: right ventricle; RVET: right ventricular ejection time; TRV: tricuspid regurgitation velocity.

**Table 5:** Bronchopulmonary Dysplasia-Associated Pulmonary Hypertension: Echo Markers.

Marker	Interpretation	Significance
PAAT < 47 ms	High PVR	Predicts PH at 36w PMA
PAAT/RVET < 0.28	Persistent PH	High sensitivity/specificity
Septal flattening	RV overload	Severe BPD

BPD: Bronchopulmonary Dysplasia; PAAT: Pulmonary Artery Acceleration Time; PAAT/RVET: Pulmonary Artery Acceleration Time to Right Ventricular Ejection Time Ratio; PH: Pulmonary Hypertension; PMA: Postmenstrual Age; RV: Right Ventricular.

**Table 6:** Congenital Diaphragmatic Hernia-Associated Pulmonary Hypertension: Echo Findings and Clinical Implication.

Domain	Echo Findings	Clinical Implication
Pulmonary tone	Severe septal flattening	Poor vasodilator response
RV function	↓TAPSE, ↓FAC	Predicts deterioration
Shunt	Fixed Right → Left PDA/PFO	Suprasystemic PH

FAC: Fractional Area Change; PDA/PFO: Patent Ductus Arteriosus/Patent Foramen Ovale; PH: Pulmonary Hypertension; RV: Right Ventricular; TAPSE: Tricuspid Annular Plane Systolic Excursion; ↓: Decrease.

**Table 7:** Adjunct and Emerging Therapies in Persistent Pulmonary Hypertension of the Newborn.

Drug	Mechanism of Action	Main Indication in PPHN	Key NPE/Echo Trigger	Precautions/Caveats
Milrinone (PDE3 inhibitor)	Increases cAMP → inotropic and lusitropic effects with pulmonary and systemic vasodilation	PPHN with RV and/or LV dysfunction, low cardiac output, or postcapillary component; inadequate response to iNO alone	Depressed RV/LV systolic function, elevated MPI, low LVOT-VTI/cardiac output, septal flattening with impaired LV filling	Risk of systemic hypotension, especially with loading dose; avoid or use cautiously in hypovolemia; monitor blood pressure and perfusion closely.
Sildenafil (PDE5 inhibitor)	Enhances NO–cGMP signaling → selective pulmonary vasodilation	Suboptimal or absent response to iNO; settings where iNO unavailable; evolving/chronic PH (e.g., BPD)	Elevated PVR with preserved or only mildly impaired LV function; persistent high PAAT/RVET abnormalities; ongoing right-to-left/bidirectional shunting despite optimized iNO	May cause systemic hypotension; use with caution in significant LV dysfunction or postcapillary PH due to risk of pulmonary edema.
Prostaglandin E1 (PGE1)	Maintains ductal patency; reduces RV afterload via right-to-left shunt “pop-off”	Severe suprasystemic PH with RV failure and closing ductus; bridge to stabilization or ECMO	Marked RV dysfunction (low TAPSE/FAC, RV dilation), septal bowing, near-systemic or suprasystemic RV pressure with restrictive ductal flow	Can worsen systemic hypotension and apnea; requires intensive monitoring; balance systemic perfusion vs. RV unloading.
Vasopressin	V1-mediated systemic vasoconstriction with relatively preserved pulmonary circulation → improves SVR and coronary perfusion	PPHN with systemic hypotension and low SVR, particularly when inotropes and vasodilators are used	Echo evidence of low systemic output with relatively preserved RV function, need to improve coronary and systemic perfusion	Excess vasoconstriction may impair end-organ perfusion; careful titration and monitoring of urine output, lactate, and extremity perfusion.
Prostacyclin analogues (e.g., epoprostenol, iloprost)	Potent pulmonary vasodilators via cAMP; anti-remodeling properties	Severe PH refractory to iNO/PDE inhibitors, including CDH- or BPD-associated PH in specialized centers	Persistently high PVR and RV strain despite optimized conventional therapy; evidence of progressive RV dysfunction	Risk of systemic hypotension and ventilation–perfusion mismatch; nebulized/continuous infusion requires expertise and close monitoring.
Bosentan (Endothelin receptor antagonist)	Blocks endothelin-1–mediated vasoconstriction and remodeling	Chronic PH, especially in BPD or CDH survivors under specialist supervision	Persistent PH on serial NPE (elevated PVR markers, RV hypertrophy/dysfunction) despite first-line oral therapy	Hepatotoxicity risk; requires liver function monitoring; limited evidence in acute PPHN and should be used in experienced centers.
Magnesium sulfate	Non-specific smooth muscle relaxant; mild pulmonary vasodilator and anti-vasospastic effects	Adjunct in refractory PH when other options limited, particularly in resource-constrained settings	Echo evidence of high PVR with preserved or moderately impaired ventricular function; used as additional vasodilator	Risk of hypotension and respiratory depression; monitor blood pressure, reflexes, and calcium.
sGC stimulators/activators (e.g., riociguat—experimental in neonates)	Direct stimulation of soluble guanylate cyclase → increases cGMP independent of NO	Emerging rescue therapy for NO-resistant PH; currently experimental and limited to research/tertiary settings	Severe PH with poor response to NO-based strategies and advanced RV dysfunction, considered in research protocols	Safety, dosing, and long-term effects in neonates not established; use restricted to clinical trials/experienced centers.
Rho-kinase (ROCK) inhibitors (experimental)	Inhibit Rho-kinase–mediated vasoconstriction and vascular remodeling	Potential option in severe, refractory PH; currently investigational	Persistent suprasystemic PH and RV failure despite maximal conventional therapy, within research framework	

BPD: bronchopulmonary dysplasia; CDH: congenital diaphragmatic hernia; ECMO: extracorporeal membrane oxygenation; FAC: fractional area change; iNO: inhaled nitric oxide; LV: left ventricle; LVOT-VTI: left ventricular outflow tract velocity time integral; MPI: myocardial performance index; PAAT: pulmonary artery acceleration time; PDE3: phosphodiesterase type 3; PDE5: phosphodiesterase type 5; PGE1: prostaglandin E1; PPHN: persistent pulmonary hypertension of the newborn; NO: nitric oxide; NPE: neonatologist-performed echocardiography; sGC: soluble guanylate cyclase; ROCK: Rho-associated protein kinase; RV: right ventricle; RVET: right ventricular ejection time; TAPSE: tricuspid annular plane systolic excursion.

## Data Availability

No new data were created or analyzed in this study. Data sharing is not applicable to this article.
